# Dexmedetomidine ameliorates liver injury and maintains liver function in patients with hepatocellular carcinoma after hepatectomy: a retrospective cohort study with propensity score matching

**DOI:** 10.3389/fonc.2023.1108559

**Published:** 2023-04-21

**Authors:** Xiaoqiang Wang, Yi-ran Li, Yumiao Shi, Xiaoying Li, Jiamei Luo, Yiqi Zhang, Bo Qi, Feixiang Wu, Yuming Sun, Zhiying Pan, Jie Tian

**Affiliations:** ^1^ Department of Anesthesiology, Renji Hospital, Shanghai Jiaotong University School of Medicine, Shanghai, China; ^2^ Department of Intensive Care Medicine, Eastern Hepatobiliary Surgery Hospital, The Third Affiliated Hospital of Naval Medical University, Shanghai, China; ^3^ Department of Anesthesiology, Eastern Hepatobiliary Surgery Hospital, The Third Affiliated Hospital of Naval Medical University, Shanghai, China

**Keywords:** hepatocellular carcinoma, liver injury, inflammation, perioperative organ damage, dexmedetomidine

## Abstract

**Background:**

Although dexmedetomidine (DEX) is widely used during the perioperative period in patients with hepatocellular carcinoma (HCC), its clinical effects on liver function and postoperative inflammation are unclear. This study aimed to explore effects of DEX on postoperative liver function and inflammation in patients with HCC after hepatectomy.

**Methods:**

A retrospective cohort study with propensity score matching was performed. A total of 494 patients who underwent hepatectomy from June 2019 to July 2020 and fulfilled the eligibility criteria were included in this study. Baseline data, liver function indexes and inflammation-related biomarkers were collected and compared between the two groups. Survival analysis was conducted to investigate the effects of DEX on the overall survival (OS) of patients. Propensity score matching (PSM) was used to minimize bias between the two groups.

**Results:**

The study cohort comprised 189 patients in the DEX-free group and 305 patients in the DEX group. Patients in the DEX group had lower levels of alanine transaminase (ALT, *P* = 0.018) and lactate dehydrogenase (LDH, *P* = 0.046) and higher level of serum albumin (ALB, *P* < 0.001) than patients in the DEX-free group before discharge. A total of 107 pairs of patients were successfully matched by PSM. Results consistently suggested that ALT and LDH levels were significantly lower (*P* = 0.044 and *P* = 0.046, respectively) and ALB levels were significantly higher (*P* = 0.002) in the DEX group than in the DEX-free group in the early postoperative period. No significant differences of inflammation-related biomarkers were observed between two groups after PSM. Neither the Kaplan–Meier survival analysis nor the multiple Cox regression survival analysis identified DEX as a contributing factor that would affect the OS of patients after PSM.

**Conclusion:**

DEX exerts protective effects on liver function while has little effects on inflammation-related biomarkers in the early postoperative period in patients undergoing hepatectomy due to HCC.

## 1 Introduction

Hepatocellular carcinoma (HCC) has one of the highest incidence rates among cancers worldwide and has become the third leading cause of death among all types of cancers (1, 2). Especially, in China, HCC is ranked as the second major cause of cancer-related death owing to the prevalence of hepatitis virus (3). It causes heavy burden on global health.

Despite the implementation of multiple treatment approaches such as ablation, liver transplantation, immune checkpoints inhibitors and CAR-T (chimeric antigen receptor T) immunotherapy for HCC in the clinic, hepatectomy remains the preferred option for patients with resectable HCC (4). Although hepatectomy effectively excises the primary tumor, it simultaneously causes unavoidable liver injury. Meanwhile, ischemia–reperfusion injury (IRI) induced by inflow occlusion during hepatectomy significantly activates local and systemic inflammatory responses, oxidative stress injury, and multiple organ injury, including the liver, kidney, and heart (4–6), which would even result in dangerous postoperative complications such as hepatic failure, renal dysfunction, and irreversible myocardial injury (4). Therefore, identifying the method to minimize liver injury and ameliorate liver function during hepatectomy will be highly clinically significant for patients with HCC.

As a highly selective α_2_-receptor agonist, dexmedetomidine (DEX) is widely used in clinical anesthesia for satisfactory sedation and analgesia without causing respiratory depression and hemodynamic instability (7). Moreover, animal and human studies have reported that DEX is effective in preventing postoperative delirium, promoting liver regeneration, inhibiting sepsis-induced systemic inflammatory response and injury, and improving the functions of important organs such as the kidney, lung, intestinal tract, and heart postoperatively or in the intensive care unit (ICU) (8–11). Several fundamental experiments have proved the protective effects of DEX on liver function after surgery by demonstrating that perioperative DEX use significantly reduced inflammatory response and oxidative stress injury in hepatectomy or liver transplantation surgeries (12–14).

Nevertheless, the effects of DEX on liver function were primarily investigated in animal studies, and only a few clinical studies with small sample sizes have investigated this issue in patients undergoing hepatectomy (6, 15, 16). Therefore, we conducted this retrospective cohort study with a suitable sample size and using propensity score matching (PSM) to determine the effects of DEX on liver function in patients undergoing surgery due to HCC.

## 2 Methods

### 2.1 Study design

This retrospective, single-center cohort study was conducted in the Eastern Hepatobiliary Surgery Hospital, Shanghai, China. This study was approved by the Eastern Hepatobiliary Surgery Hospital’s Institutional Review Board (Number: EHBHKY2021-K-011). We included only those patients who granted authorization for future research use of their medical records. This study was conducted according to the Declaration of Helsinki and was consistent with the STROBE criteria.

### 2.2 Participants

Patients aged >18 years, with American Society of Anesthesiologists (ASA) scores of I–III and Child–Pugh stages A and B, and who underwent elective hepatectomy for HCC treatment from June 2019 to July 2020 were included in this study. The exclusion criteria were as follows: (1) malignant tumors in other organs, (2) a combination of thermal ablation or chemoablation during hepatectomy, (3) any congenital liver disease (e.g., polycystic liver disease and Wilson’s disease) or autoimmune liver disease, (4) liver failure before surgery [defined according to guidelines (17)], and (5) other severe organ failure before surgery (i.e., heart failure was defined as a left ventricular ejection fraction of < 35%, and renal failure was defined as a serum creatinine level of >442 μmol/L) (18, 19).

### 2.3 Intervention, anesthesia, and surgical anesthesia care

Patients were divided into the DEX or DEX-free group based on whether they received intravenous DEX or not during the perioperative period. As DEX is not available in the ICU or wards in the Eastern Hepatobiliary Surgery Hospital, patients in the DEX group were infused with DEX only during the surgery. The dosage of DEX was collected from the digital medical records system.

Patients in both groups underwent hepatectomy under general anesthesia. They were monitored according to the ASA monitoring standards. Based on the preference of anesthetists, patients received propofol, midazolam (optional), fentanyl/sufentanil/oxycodone, and rocuronium for anesthesia induction. General anesthesia was maintained with sevoflurane, rocuronium, sufentanil/remifentanil, propofol (optional), and DEX (optional). Mechanical ventilation was initiated after tracheal intubation, and P_ET_CO_2_ was maintained in the range of 35–45 mmHg. The mean arterial blood pressure was maintained at >60 mmHg with an infusion of Ringer’s lactate solution and artificial colloid, or vasoactive agents when needed, during the operation. Blood transfusion was initiated when the patient’s hemoglobin level decreased to <7 mg/dl or decided by the anesthesiologists based on the patients’ age, hemodynamic stability, and presurgical hemoglobin levels when the hemoglobin level was 7–10 mg/dl.

For patients who received DEX during surgery, DEX was diluted in 0.9% saline to a final concentration of 4 μg/ml, and a total dose of 0.5–1.0 μg/kg was injected through an intravenous pump during anesthesia induction and maintenance, as determined by the anesthesiologist’s preference.

Standard hepatectomy was performed with or without temporary hepatic inflow occlusion by experienced liver surgeons. The duration of hepatic inflow occlusion was determined by surgeons. The same therapy guidelines were followed by all surgical teams.

Patients were transferred to the ICU or recovered in the post-anesthesia care unit after surgery, as decided by surgeons. Postoperative analgesia was provided by patient-controlled intravenous analgesia (PCIA) based on a consensus between the patient and the clinical team. For PCIA, an intravenous pump with 2.0 μg/kg sufentanil and 100 mg flurbiprofen axetil in 100 ml normal saline was used. The infusion rate was 2 ml/h with 15-min block time. In general, the pump was maintained for the first 2 days after the operation.

### 2.4 Variables and data sources

Preoperative clinical characteristics of the patients, including gender, age, height, weight, ASA score, Child–Pugh stage, TNM stage, and comorbidity, were recorded. Intraoperative and postoperative factors, including tumor location, tumor size, tumor number, surgery information, types and doses of intraoperative anesthetics, blood transfusion, fluid balance, postoperative analgesia, ICU stay, and postoperative complications, were also collected. Tumor size was defined as the maximum diameter of the tumor or the average of the maximum diameter when there are more than one tumor. The expression levels of liver function biomarkers, including serum alanine transaminase (ALT), aspartate aminotransferase (AST), lactate dehydrogenase (LDH), total bilirubin (TBIL), and serum albumin (ALB), were collected at three time points: before operation, 24 h after operation, and before discharge (the latest biochemical detection before discharge from the hospital). The levels of inflammation biomarkers, including serum C-reactive protein (CRP), WBC, and percentage of neutrophils (N%) in peripheral blood, were also collected at the abovementioned three time points.

All data were retrieved from the digital medical system or paper medical records. Two trained researchers completed the data collection and entered the data into the Excel or EpiData system. Data regarding the survival condition of patients were obtained from the digital medical system, surgeons, or telephone follow-up. Data were censored for patients who were alive at the follow-up closure date (April 7, 2022).

### 2.5 Study outcomes

The primary outcome was the serum ALT level. The secondary outcomes included the expression levels of inflammatory biomarkers (serum CRP, WBC number, and N%); the serum levels of AST, TBIL, LDH, and ALB; and the overall survival (OS) of the patients.

### 2.6 Statistical analysis

Statistical analyses were conducted using IBM SPSS Statistics 23.0 (SPSS Inc., Armonk, NY, USA). Categorical variables were expressed as number (n), and continuous variables were expressed as mean ± standard deviation or mean ± standard error (SEM) or median [25% interquartile range, 75% interquartile range] based on normality. Student’s *t*-test or the Mann–Whitney U test was conducted to compare continuous variables. Categorical variables were compared using the χ2 test or Fisher’s exact test, where appropriate. The differences in the levels of serum biomarkers reflecting liver function and inflammation were analyzed using two-way repeated analysis with the Bonferroni correction. All statistical tests were two-sided, and a *P* value of <0.05 was considered statistically significant.

The Kaplan–Meier survival analysis was performed, and curves were generated using the log-rank test to identify the differences in OS between the two groups. The curve for cumulative risk was also generated. Next, a multivariable Cox regression analysis was conducted to adjust potential bias. Potential risk factors with *P* < 0.05 in the univariable Cox analyses were included in the multivariable Cox regression analysis.

The PSM method was applied to eliminate potential bias between the two groups. A logistic regression model of PSM was constructed using the covariates of ASA score, TNM stage, viral hepatitis, cirrhosis, TACE before surgery, tumor size, duration of hepatic inflow occlusion, volume of bleeding, plasma transfusion, RBC transfusion, volume of crystalloid fluid, volume of colloidal fluid, dosage of midazolam, and dosage of NSAIDs. We applied 1:1 nearest neighbor matching without replacement to ensure that conditional bias was minimized. The caliper width was 0.1.

## 3 Results

**Figure 1:**
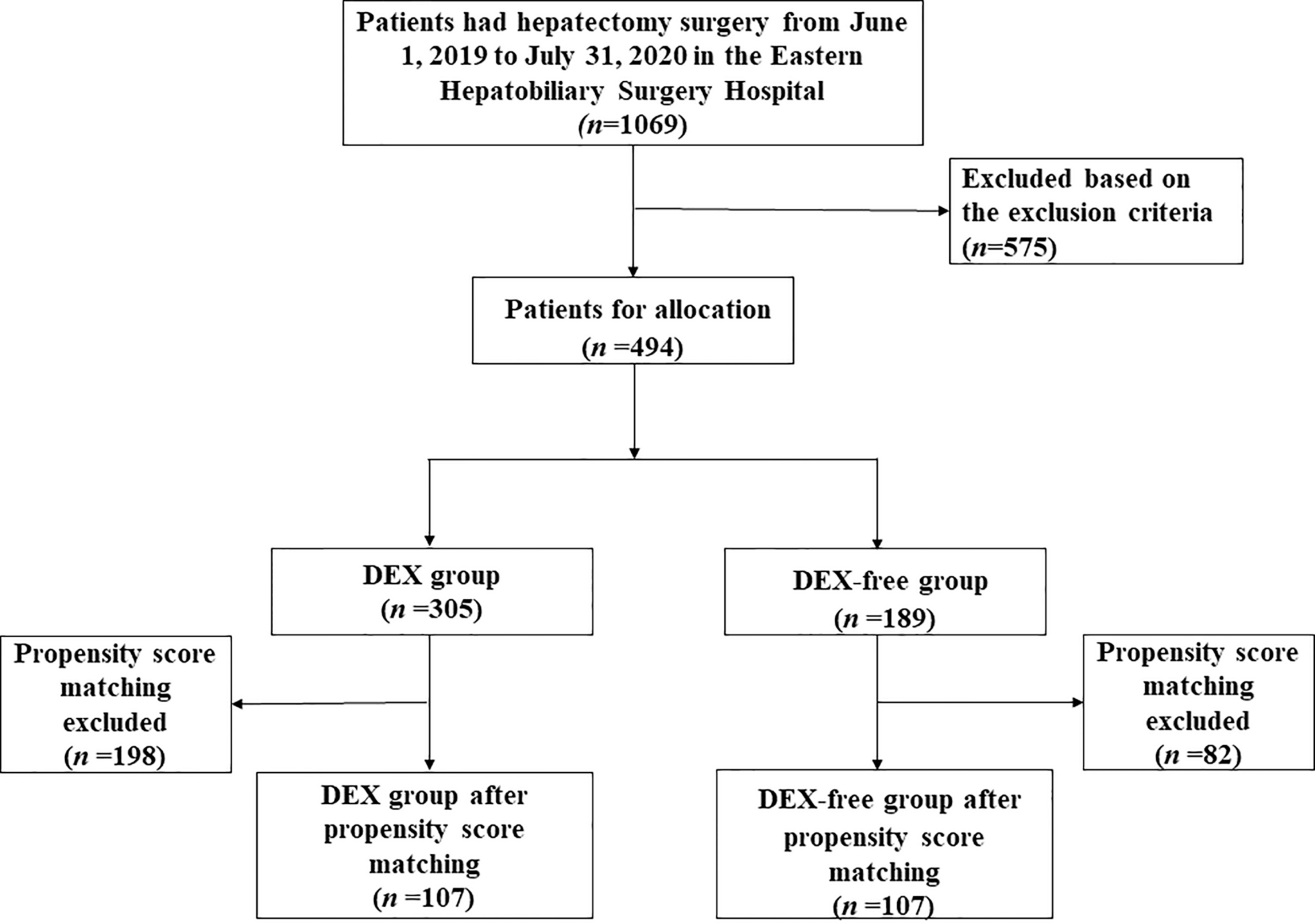
Flow diagram detailing the selection process for patients in this study.

As depicted in **Figure 1**, a total of 1069 patients who underwent hepatectomy from June 1, 2019, to July 31, 2020, were screened for the study. Then, 494 who fulfilled the criteria were finally enrolled and divided into the DEX group (*n* = 305) and DEX-free group (*n* = 189). In addition to DEX dosage, several other baseline characteristics of the patients, including the ASA score, hepatic inflow occlusion duration, volume of bleeding, and midazolam dosage, were significantly different between the two groups, as shown in **Table 1**. To eliminate bias between the two groups, PSM was performed, and 107 pairs of patients were successfully matched finally. No significant differences were observed in the PSM score and baseline characteristics, except the dosage of DEX, between the two groups after PSM (**Table 2**).

**Table 1:** Clinical characteristics of patients between two groups before PSM.

	DEX-free group (n=189)	DEX group (n=305)	*P* value
Preoperative			
Gender (male/female)	153/36	246/59	0.94
Age (year)	57.1 (10.6)	56.1 (11.2)	0.32
Height (cm)	167.1 (6.9)	168.0 (6.1)	0.15
Weight (kg)	67.6 (11.5)	67.3 (9.0)	0.77
ASA stage*			
I and II	182 (97.3%)	241 (79.8%)	**0.00**
III	5 (2.7%)	61 (20.2%)	
Child-Pugh stage (A/B)	189/0	304/1	1.00
TNM stage*			
I	103 (54.5%)	169 (56.5%)	0.53
II	77 (40.7%)	110 (36.8%)	
III and IV	9 (4.8%)	20 (6.7%)	
Hypertension (Yes/No)	40/149	75/230	0.38
Diabetes (Yes/No)	21/168	37/268	0.73
Smoking (Yes/No)	71/118	139/166	0.08
Alcohol drinking (Yes/No)	56/133	91/214	0.96
Viral hepatitis*^§^ (Yes/No)	145/44	238/54	0.20
HBV-DNA ≥ 50IU/ml* (Yes/No)	74/111	132/171	0.44
Cirrhosis* (Yes/No)	102/86	158/147	0.60
PVTT* (Yes/No)	7/167	18/225	0.15
TACE before surgery (Yes/No)	32/157	40/265	0.24
Intraoperative			
Open/laparoscopic	182/7	290/14	0.63
Left/right/caudate/left + right lobe resection	49/121/2/17	60/206/6/32	0.37
Tumor number (Single/Multiple)	163/26	258/46	0.67
Tumor size (cm)	5.3 (3.7)	5.8 (4.0)	0.18
Length of hepatic inflow occlusion (min)	16 [9, 22]	20 [6, 33]	**0.00**
Volume of bleeding (ml)	200 [200, 400]	300 [200, 500]	**0.00**
Plasma transfusion (Yes/No)	17/172	67/238	**0.00**
RBC transfusion (Yes/No)	17/172	63/242	**0.00**
Crystalloid fluid** (ml)	1500 [1000, 1500]	1000 [1000, 1500]	**0.00**
Colloid fluid** (ml)	500 [500, 500]	700 [500, 1000]	**0.00**
ALB transfusion (g)	20 [20, 20]	20 [20, 20]	0.71
Dosage of opioids (equivalent dose of morphine, mg)	159.4 (91.3)	171.3 (97.2)	0.17
NSAIDs^§§^ (mg)	0 [0, 37.5]	0 [0, 0]	0.18
Midazolam (mg)	2.0 [2.0, 2.0]	2.0 [0.0, 3.0]	**0.00**
DEX (μg)	0	40 [40, 50]	**0.00**
Postoperative			
ICU care (Yes/No)	46/142	88/214	0.26
PCIA (Yes/No)	125/64	212/93	0.43
Postoperative complications			
Fever^§§§^ (> 38°C over 48 h)	1 (0.5%)	3 (1.0%)	0.54
Pain	14 (7.4%)	18 (5.9%)	
Bleeding	2 (1.1%)	2 (0.7%)	
Severe PONV^§§§^	1 (0.5%)	0 (0.0%)	

Variables are shown as “mean (SD)”, “number (%)” or “median [25% quartile, 75% quartile]”. HCC, Hepatocellular carcinoma; DEX, dexmedetomidine; ASA, American Society of Anesthesiologists; TNM, Clinicopathological stage; HBV, hepatitis B viral; PVTT, portal vein tumor thrombus; TACE, transcatheter arterial chemoembolization; RBC, red blood cell; ALB, Albumin; NSAIDs, non-steroidal anti-inflammatory drugs; PONV, postoperative nausea and vomiting; ICU, intensive care unit; PCIA, patient-controlled intravenous analgesia; SD, standard deviation. PSM, propensity score matching.

Bold values mean *P* < 0.05 *Factors with single asterisk indicates patients with missing data.

^§^Viral hepatitis includes HBV and HCV infection.

**Crystalloid fluid means lactated Ringer’s solution and colloid fluid means hydroxyethyl starch solution (Voluven) in the studied center.

^§§^NSAIDs means flurbiprofen axetil in the studied center.

^§§§^ The body temperature is reflected by the armpit temperature. Severe PONV is defined as episodes of expulsion of gastric contents that need antiemetic treatment.

**Table 2:** Clinical characteristics of HCC patients between two groups after PSM.

	DEX-free group (n=107)	DEX group (n=107)	*P* value
Propensity score	0.49 (0.23)	0.44 (0.23)	0.11
Preoperative			
Gender (male/female)	92/15	87/20	0.36
Age (year)	55.9 (10.6)	54.8 (11.1)	0.49
Height (cm)	167.9 (6.8)	168.0 (5.5)	0.96
Weight (kg)	68.1 (11.4)	67.7 (9.0)	0.77
ASA stage			
I and II	102 (95.3%)	94 (87.9%)	0.09
III	5 (4.7%)	13 (12.1%)	
Child-Pugh stage (A/B)	107/0	107/0	1.00
TNM stage			
I	60 (56.1%)	57 (53.3%)	0.89
II	43 (40.2%)	45 (42.1%)	
III and IV	4 (3.7%)	5 (4.7%)	
Hypertension (Yes/No)	19/88	22/85	0.60
Diabetes (Yes/No)	13/94	15/92	0.69
Smoking (Yes/No)	41/66	42/65	0.89
Alcohol drinking (Yes/No)	31/76	32/75	0.88
Viral hepatitis^§^ (Yes/No)	87/20	85/22	0.73
HBV-DNA ≥ 50IU/ml* (Yes/No)	46/61	45/62	0.89
Cirrhosis (Yes/No)	55/52	54/53	0.89
PVTT (Yes/No/Missing)	4/96/7	6/83/18	0.40
TACE before surgery (Yes/No)	20/87	17/90	0.59
Intraoperative			
Open/laparoscopic	105/2	99/8	0.10
Left/right/caudate/left + right lobe resection	28/70/1/8	24/74/2/7	0.85
Tumor number (Single/Multiple)	94/13	91/16	0.55
Tumor size (cm)	5.4 (4.0)	5.5 (3.7)	0.95
Length of hepatic inflow occlusion (min)	17 [9, 23]	15 [0, 29]	0.97
Volume of bleeding (ml)	200 [200, 400]	300 [200, 400]	0.41
Plasma transfusion (Yes/No)	13/94	18/89	0.33
RBC transfusion (Yes/No)	13/94	18/89	0.33
Crystalloid fluid* (ml)	1500 [1000, 1500]	1000 [1000, 1500]	0.56
Colloid fluid* (ml)	500 [500, 500]	500 [500, 750]	0.40
ALB transfusion (g)	20 [20, 20]	20 [20, 20]	0.18
Dosage of opioids (equivalent dose of morphine, mg)	157.8 (90.5)	142.8 (90.8)	0.23
NSAIDs^§§^ (mg)	0 [0, 0]	0 [0, 0]	0.43
Midazolam (mg)	2.0 [2.0, 2.0]	2.0 [2.0, 3.0]	0.45
DEX (μg)	0	40 [40, 40]	0.00
Postoperative			
ICU care (Yes/No)	30/76	34/70	0.49
PCIA (Yes/No)	68/39	67/40	0.89
Postoperative complications			
Fever^§§§^ (> 38°C over 48 h)	0 (0.0%)	1 (0.9%)	0.70
Pain	8 (7.5%)	8 (7.5%)	
Bleeding	2 (1.9%)	1 (0.9%)	
Severe PONV^§§§^	0 (0.0%)	0 (0.0%)	

Variables are shown as “mean (SD)”, “number (%)” or “median [25% quartile, 75% quartile]”. HCC, Hepatocellular carcinoma; DEX, dexmedetomidine; ASA, American Society of Anesthesiologists; TNM, Clinicopathological stage; HBV, hepatitis B viral; PVTT, portal vein tumor thrombus; TACE, transcatheter arterial chemoembolization; RBC, red blood cell; ALB, Albumin; NSAIDs, non-steroidal anti-inflammatory drugs; PONV, postoperative nausea and vomiting; ICU, intensive care unit; PCIA, patient-controlled intravenous analgesia; SD, standard deviation; PSM, propensity score matching.

^§^Viral hepatitis includes HBV and HCV infection.

*Crystalloid fluid means lactated Ringer’s solution and colloid fluid means hydroxyethyl starch solution (Voluven) in the studied center.

^§§^NSAIDs means flurbiprofen axetil in the studied center.

^§§§^ The body temperature is reflected by the armpit temperature. Severe PONV is defined as episodes of expulsion of gastric contents that need antiemetic treatment.

### 3.1 Primary endpoint

**Figure 2:**
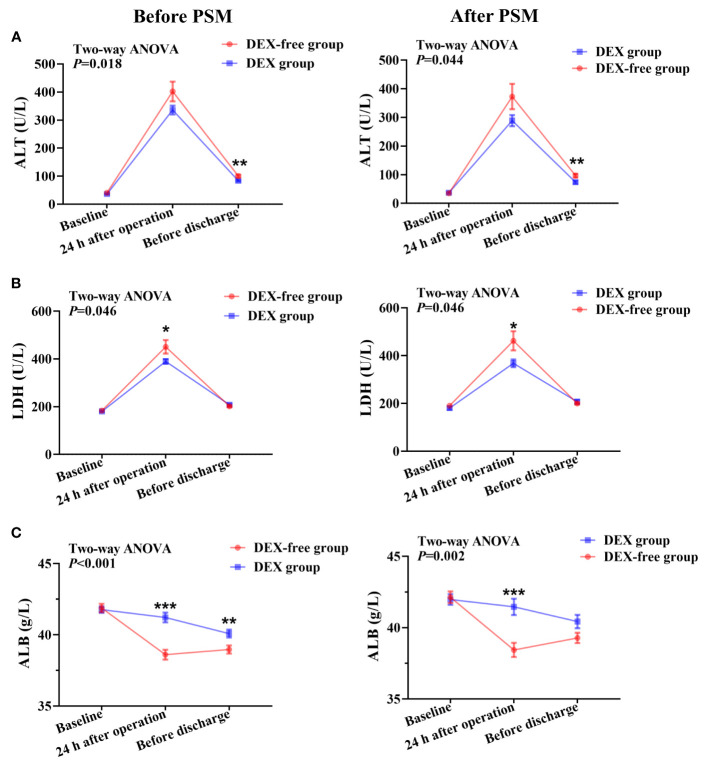
Serum levels of biomarkers of liver function at various time points in patients undergoing hepatectomy before and after PSM. **(A)**, serum ALT levels; **(B)**, serum LDH levels; **(C)**, serum ALB levels. Data were expressed as mean ± standard error. *, *P* < 0.05; **, *P* < 0.01; ***, *P* < 0.001.

Two-way repeated analysis of serum ALT levels revealed no difference in ALT levels between the two groups before surgery (**Figure 2A**). Although the ALT levels in both groups were generally higher postoperatively than preoperative baseline levels, the levels were significantly lower in the DEX group than in the DEX-free group after surgery (*P* = 0.018 before PSM and *P* = 0.044 after PSM, **Figure 2A**). *Post hoc* analysis further showed that the difference between the two groups was significant before discharge (*P* = 0.003 before PSM and *P* = 0.005 after PSM), but not at 24 h after surgery, although the difference appeared to be greater at this time point (**Figure 2A**).

### 3.2 Effects of DEX on other liver function biomarkers

**Figure 3:**
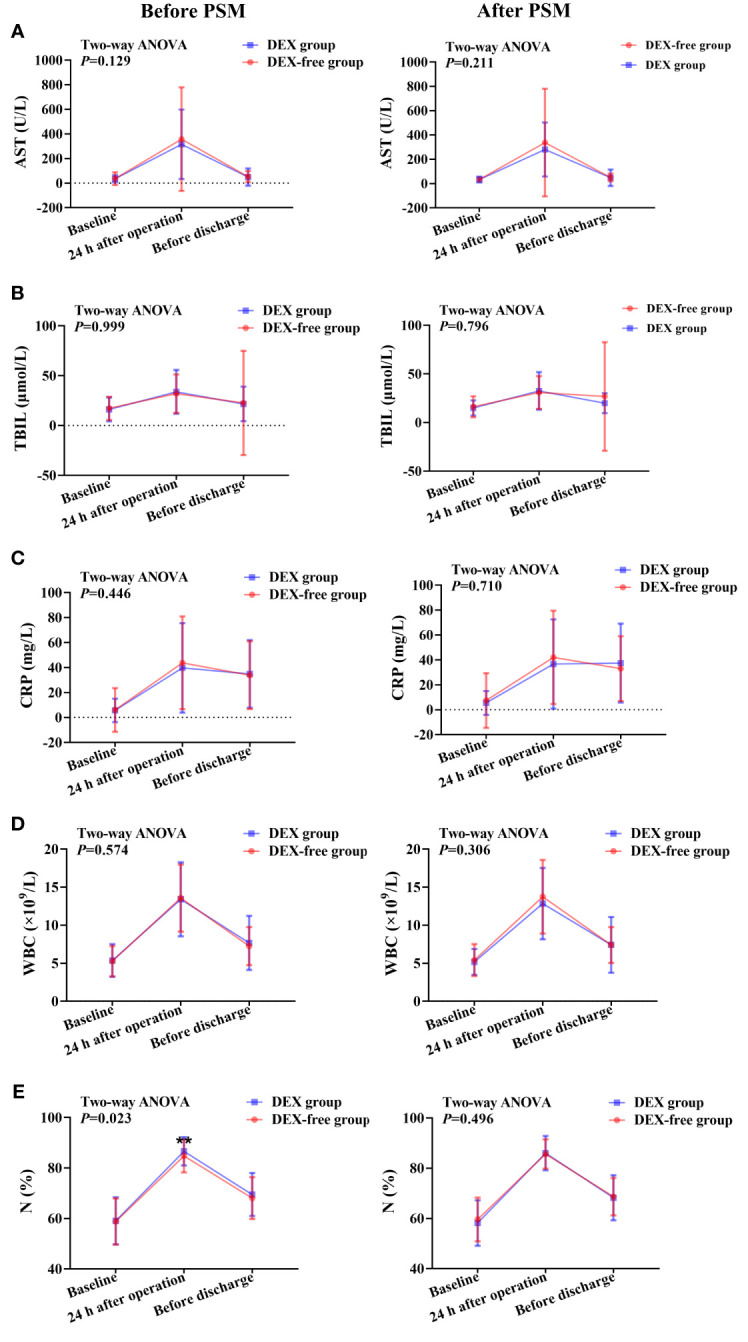
Expression levels of biomarkers of liver function and inflammation at various time points in patients undergoing hepatectomy before and after PSM. **(A)**, serum AST levels; **(B)**, serum TBIL levels; **(C)**, serum CRP levels; **(D)**, peripheral WBC count; **(E)**, percentage of neutrophils (N%) in peripheral blood. Data were expressed as mean ± standard deviation. **, *P* < 0.01.

The serum levels of AST, LDH, TBIL, and ALB showed no differences between the two groups before surgery (**Figures 2**, **3**). As shown in **Figure 2B**, the LDH serum levels were significantly lower in the DEX group than in the DEX-free group postoperatively (*P* = 0.046 before PSM and *P* = 0.046 after PSM). Interestingly, the serum levels of AST and TBIL remained comparable between the two groups at all the examined time points both before and after PSM (**Figures 3A, B**).

The results also revealed that the ALB serum levels were higher in the DEX group than in the DEX-free group after surgery (**Figure 2C**). The difference was more obvious at 24 h after surgery (*P* < 0.001 for both before and after PSM) and remained significant till before discharge (*P* = 0.008 before PSM and *P* = 0.054 after PSM). This finding indicated that DEX might not only alleviate liver injury but also maintain the productive function of the liver.

### 3.3 Effects of DEX on inflammation-related biomarkers

Although there were no differences in serum CRP levels and peripheral WBC count between the two groups at all time points (**Figures 3C, D**), the N% in peripheral blood was slightly but significantly higher in the DEX group at 24 h after surgery than in the DEX-free group before PSM (*P* = 0.001, **Figure 3E**). However, the difference was absent between the two groups after PSM (*P* = 0.496, **Figure 3E**). These results suggested that DEX did not have much influence on postoperative inflammation in patients undergoing hepatectomy.

### 3.4 Effects of DEX on the OS of patients

**Figure 4:**
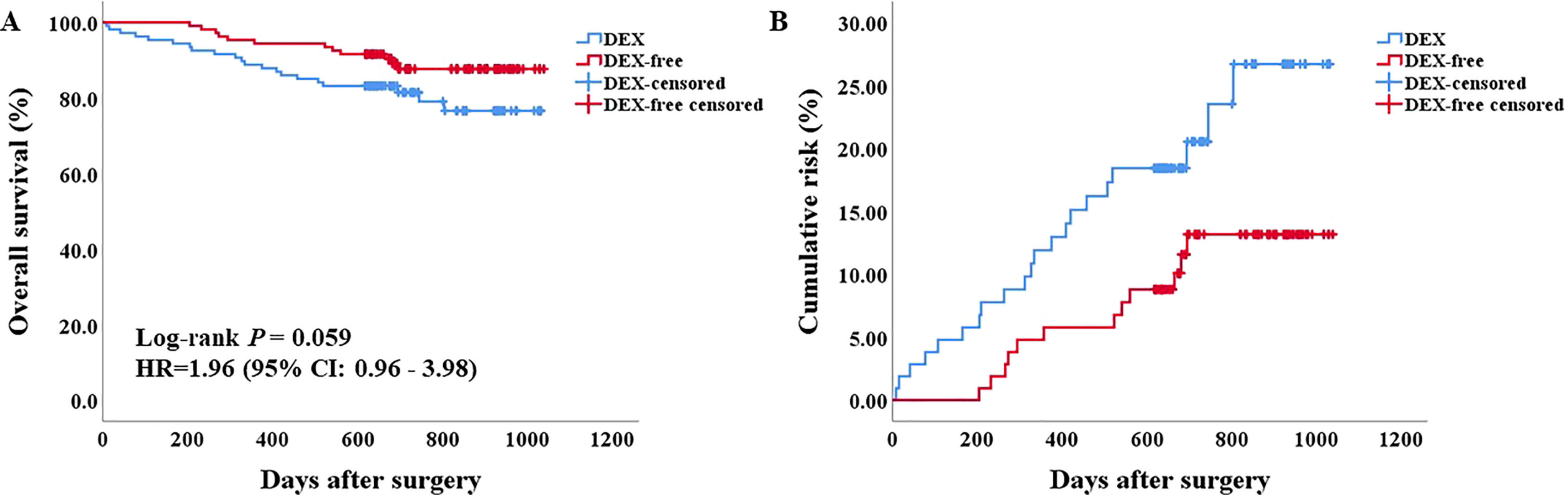
Survival analysis of patients after PSM. **(A)** the Kaplan–Meier survival curve of patients in the two groups. **(B)** the cumulative risk of patients in the two groups.

Before PSM, both univariable and multivariable Cox regression analyses of OS suggested a worse OS for patients in the DEX group (**Supplementary Table 1**, **Supplementary Figure 1**). However, there was no difference in the OS of patients between the two groups (*P* = 0.059, HR = 1.96, 95% CI: 0.96–3.98) as evaluated by the Kaplan–Meier survival analysis after PSM (**Figure 4**). We next included all the risk factors which *P* < 0.05 in univariable Cox analyses into a multivariable Cox regression analysis to adjust potential bias. As shown in **Table 3**, there was still no difference in the OS between the two groups (*P* = 0.076, HR = 2.00, 95% CI: 0.93–4.29). Interestingly, the multiple Cox regression analysis suggested that drinking, PVTT, and tumor size were independent risk factors for the OS of patients undergoing hepatectomy due to HCC.

**Table 3:** Univariable and multivariable Cox regression model analysis of OS in patients after PSM.

Independent predictive factor	Univariable Cox analysis			Multiple Cox analysis			
	HR	95% CI	*P* value	HR		95% CI	*P* value
DEX usage							
DEX-free	1	0.96-3.98	0.059	1		0.93-4.29	0.076
DEX	1.96			2.00			
Drinking							
No	1	1.02-4.00	0.045	1		1.05-4.78	0.038
Yes	2.02			2.23			
PVTT							
No	1	2.45-14.86	0.000	1		1.49-10.12	0.005
Yes	6.04			3.89			
Tumor size*							
< 3 cm	1	1.13-3.17	0.015	1		1.01-4.27	0.048
≥ 3 cm	1.89			2.07			
Hepatic inflow occlusion							
No	1	0.24-0.96	0.039	1		0.24-1.27	0.165
Yes	0.48			0.56			
Plasma or RBC transfusion							
No	1	1.07-4.75	0.032	1		0.62-3.79	0.358
Yes	2.26			1.53			

OS, overall survival; DEX, dexmedetomidine; HR, hazard ratio; CI, confidence interval; PVTT, portal vein tumor thrombus; RBC, red blood cell; PSM, propensity score matching.

* Tumor size is defined as the maximum diameter of tumor or the sum of maximum diameter when tumor number exceeds one.

## 4 Discussion

This retrospective cohort study suggests possible protective effects of DEX on liver function in patients with HCC who underwent hepatectomy. Perioperative DEX use may not only reduce liver injury but also improve the liver function of producing ALB during hepatectomy. Little effects of DEX on early postoperative inflammation were found between two groups.

With the development of surgical techniques and perioperative management, resection of hepatic tumors has been one of the most popular choices for patients with HCC (2018). The application of the occlusion of portal triad and total vascular exclusion minimizes intraoperative blood loss and the need for blood transfusion (4, 20). Nonetheless, both techniques cause inevitable IRI that may impair liver function and regeneration after hepatectomy. Furthermore, surgical trauma and stress response, excessive inflammatory response, and poor liver conditions with hepatitis or cirrhosis cause heavy burden on the liver (21). Therefore, perioperative protection of the liver is of significant concern for patients undergoing hepatectomy (22).

Numerous strategies have been designed for reducing liver injury and postoperative inflammatory response, and preserving liver function during hepatectomy (23, 24). For instance, studies have suggested that remote ischemia preconditioning (RIPC) could effectively reduce hepatic IRI after liver resection. In a randomized controlled trial (RCT) (23), our team investigated the effects of RIPC on hepatic IRI in patients undergoing liver resection (23). It was observed that the serum levels of ALT and AST were significantly decreased in the RIPC group compared to those in the control group. Second, some promising drugs such as ulinastatin and oxygen radical scavengers have been used for inhibiting inflammatory responses and oxidative stress (25, 26). However, their clinical application and validation are rarely reported and require more evidence.

DEX, a widely used sedative during surgery, was approved for sedation and analgesia by the United States Drug and Food Administration in 1999 (7). Owing to its excellent advantages of sedation, analgesia, antianxiety, inhibition of sympathetic nervous excitation, cardiovascular stabilization, and prevention of postoperative delirium, DEX has been widely used in clinical anesthesia and the ICU (8, 10, 27, 28). The contraindications of DEX mainly include 1) patients allergic to DEX, 2) pregnant, lactating women, and patients with severe heart block. Interestingly, numerous basic studies have also shown that DEX exerts strong multiorgan protective effects, including the liver, lung, heart, kidney, brain, and intestinal tract (5, 29, 30). These exciting findings prompt a series of clinical investigations. A meta-analysis discussed the effects of DEX on attenuating one-lung ventilation-associated lung injury by reviewing 20 clinical trials (31). The results suggested that perioperative administration of DEX could attenuate inflammation and ameliorate pulmonary oxygenation. Another meta-analysis reported that perioperative DEX infusion inhibited the release of epinephrine, norepinephrine, and cortisol and decreased the levels of blood glucose, interleukin (IL)-6, tumor necrosis factor-α (TNF-α), and CRP (5). In addition, the immune function was improved (5, 28, 32). Li et al. (27) also systematically reviewed the anti-inflammatory effects of perioperative DEX administration as an adjunct to general anesthesia in 15 clinical trials and reported significant decreases in the serum levels of IL-6, IL-8, and TNF-α after DEX use.

Several clinical studies have also explored the effects of DEX on liver protection (15, 16, 29). In an RCT conducted by Wang et al., perioperative administration of DEX was found to attenuate intestinal and hepatic injury in patients undergoing elective liver resection with inflow occlusion with no potential risk (15). In another RCT conducted by Zhang et al., the concentrations of α-glutathione S-transferase, IL-6, TNF-α, ALT, and AST were found to be significantly lower in the DEX group than in the control group (6). Nevertheless, the sample sizes of these studies were relatively small (*n* = 22–29 per group). In our study, we included 494 patients for analysis in total. Protective effects of DEX on the liver along with a decrease in the serum levels of ALT and LDH in the DEX group were observed, compared to those in the DEX-free group. Interestingly, our results also suggested that the ALB level was significantly higher at 24h after surgery in the DEX group, which had not been reported previously in the case of hepatectomy. It appears that DEX could maintain the productive function of the liver as well. These findings support the use of DEX for reducing liver injury and maintaining ALB production in hepatectomy, which may bring significant improvement of liver function for patients. However, no differences in the levels of inflammation biomarkers such as serum CRP, WBC, and N% in peripheral blood between the two groups were found, indicating that DEX may exerted limited effects on early postoperative inflammatory responses.

The potential mechanism of action of DEX in alleviating liver injury involves multiple aspects such as anti-inflammatory and anti-IRI effects (33, 34), inhibition of hepatocyte apoptosis (35, 36), promotion of liver regeneration (9), regulation of immune function, and attenuation of oxidative stress (13, 35, 37). For instance, Zhang et al. reported that DEX could alleviate hepatic injury following intestinal IRI *in vivo* and *vitro* by upregulating β-catenin expression (34). Zhao et al. found that DEX alleviated hepatic injury by inhibiting oxidative stress and activating the Nrf2/HO-1 signaling pathway *in vitro* (13). Other potential signaling pathways that are regulated by DEX include TLR4/MyD88/NF-κB, GSK-3β/MKP-1/Nrf2, and PI3K/AKT (35, 38, 39). Studies have also shown that miRNA and LncRNA were regulated by DEX (36, 40, 41).

Regarding the effect of DEX on cancer biology, it still remains unclear and controversial (41–44). Basic and clinical studies have suggested that DEX could regulate the malignancy of cancer cells and influence the prognosis of patients, but the conclusions are conflicting (41, 44, 45). Though significant decrease in OS was found in the DEX group compared with the DEX-free group only before PSM, the difference disappeared after PSM. Therefore, it is hard to draw conclusions regarding the effects of DEX on the prognosis of patients with HCC based on the present findings, and therefore further well-designed, large sample size, prospective studies are required to explore the effects of DEX on the malignance of cancer cells. Considering the possible adverse effects of DEX on the long-term prognosis of patients with HCC, we should balance the benefits and harm of DEX for patients undergoing hepatectomy.

This study has several limitations. First, it is a single-center retrospective study, and a multicenter or a prospective cohort study with a larger sample size would elevate the reliability of our findings. Second, the duration of protective effects induced by DEX is unclear, and more investigative time points are necessary. Third, examination of more liver function and inflammation biomarkers may help us understand the effects of DEX on liver function and inflammatory response in a more comprehensive manner.

## Data availability statement

The original contributions presented in the study are included in the article/**Supplementary Material**. Further inquiries can be directed to the corresponding authors.

## Ethics statement

The studies involving human participants were reviewed and approved by This study was approved by the Eastern Hepatobiliary Surgery Hospital’s Institutional Review Board (Number: EHBHKY2021-K-011). The patients/participants provided their written informed consent to participate in this study.

## Author contributions

Conceptualization, XW, YSu and JT. Methodology, XW, BQ and Y-RL. Software, XW and JL. Validation, YSh, ZP and YSu. Formal analysis, XW, ZP and FW. Investigation, JT. Resources, XW, XL and YL. Data curation, XW, YZ and XL. Writing—original draft preparation, XW and YSh; writing—review and editing, ZP and JT; supervision, JT. Funding acquisition, YSu, ZP, BQ and JT. All authors contributed to the article and approved the submitted version.

## Supplementary material

Supplementary Figure 1 Survival analysis of patients before PSM. **(A)**, the Kaplan–Meier survival curve of patients in the two groups. **(B)**, the cumulative risk of patients in the two groups.
