# Supplementary material for: Dexmedetomidine ameliorates liver injury and maintains liver function in patients with hepatocellular carcinoma after hepatectomy: a retrospective cohort study with propensity score matching
Source: Front Oncol. 2023 Apr 21;13:1108559. doi: 10.3389/fonc.2023.1108559 (PMC10160666; doi:10.3389/fonc.2023.1108559)
Supplement: Supplementary file 1 [file Table_1.docx]

Supplementary Table 1. Univariable and multivariable Cox regression model analysis of OS in patients before PSM.

| Independent predictive factor | Univariable Cox analysis | | | | Multivariable Cox analysis | | |
| --- | --- | --- | --- | --- | --- | --- | --- |
|  | HR | 95% CI | *P* value | HR | | 95% CI | *P* value |
| DEX usage  DEX-free  DEX | 1  1.72 | 1.06-2.79 | 0.028 | 1  1.80 | | 1.03-3.14 | 0.039 |
| Viral hepatitis  No  Yes | 1  0.59 | 0.36-0.96 | 0.033 | 1  0.51 | | 0.29-0.90 | 0.021 |
| HBV-DNA ≥ 50IU/ml |  |  |  |  | |  |  |
| No | 1 |  |  | 1 | |  |  |
| Yes | 1.57 | 1.01-2.44 | 0.043 | 1.69 | | 0.99-2.87 | 0.054 |
| TNM stage |  |  |  |  | |  |  |
| Ⅰ and Ⅱ | 1 |  |  | 1 | |  |  |
| Ⅲ and Ⅳ | 3.07 | 1.62-5.80 | 0.001 | 1.96 | | 0.92-4.20 | 0.083 |
| PVTT  No  Yes | 1  3.82 | 2.00-7.29 | 0.000 | 1  2.45 | | 1.20-5.00 | 0.014 |
| Tumor size*  < 3 cm  ≥ 3 cm | 1  3.57 | 1.55-8.20 | 0.003 | 1  1.09 | | 1.03-1.15 | 0.005 |
| PCIA |  |  |  |  | |  |  |
| No | 1 |  |  | 1 | |  |  |
| Yes | 4.12 | 2.06-8.24 | < 0.001 | 2.57 | | 1.02-6.47 | 0.046 |
| Plasma or RBC transfusion  No  Yes | 1  3.97 | 2.57-6.15 | < 0.001 | 1  2.34 | | 1.34-4.06 | 0.003 |

OS, overall survival; DEX, dexmedetomidine; HR, hazard ratio; CI, confidence interval; PVTT, portal vein tumor thrombus; RBC, red blood cell; PSM, propensity score matching.

* Tumor size is defined as the maximum diameter of tumor or the sum of maximum diameter when tumor number exceeds one.
