# Supplementary figures and images for: Dexmedetomidine ameliorates liver injury and maintains liver function in patients with hepatocellular carcinoma after hepatectomy: a retrospective cohort study with propensity score matching
Source: Front Oncol. 2023 Apr 21;13:1108559. doi: 10.3389/fonc.2023.1108559 (PMC10160666; doi:10.3389/fonc.2023.1108559)

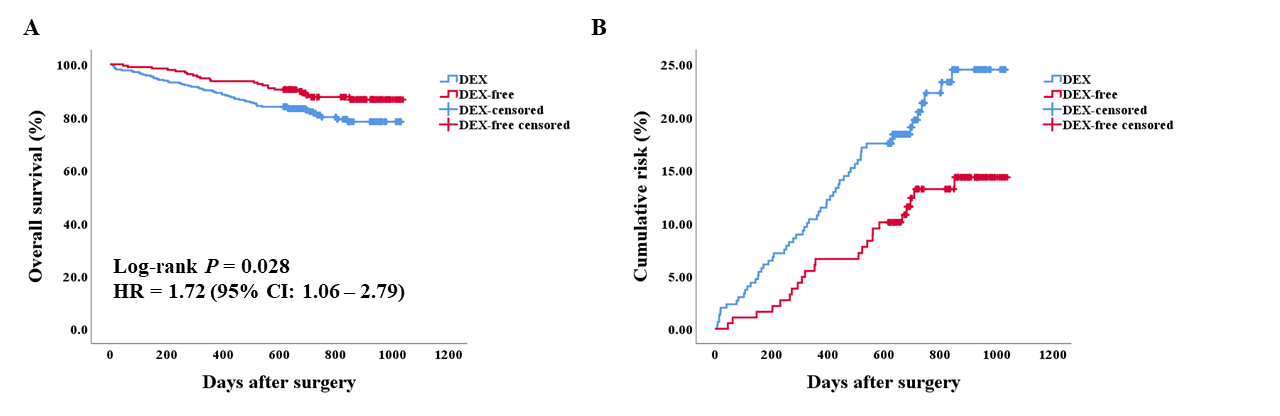

Supplement: Supplementary Figure 1 — Survival analysis of patients before PSM. (A), the Kaplan–Meier survival curve of patients in the two groups. (B), the cumulative risk of patients in the two groups. [file Image_1.tif]
